# Comparing guided, dialogic, and sign language instruction in deaf and hard-of-hearing students' narrative comprehension

**DOI:** 10.3389/fpsyg.2026.1982563

**Published:** 2026-10-20

**Authors:** Necla Işıkdoğan Uğurlu

**Affiliations:** Department of Special Education, Faculty of Education, Zonguldak Bulent Ecevit University, Zonguldak, Turkiye

**Keywords:** deaf and hard-of-hearing students, dialogic reading, guided reading, mixed methods, narrative comprehension, sign language instruction, story mapping

## Abstract

**Purpose:**

This study compared guided reading, dialogic reading, and sign language instruction for improving narrative comprehension among deaf and hard-of-hearing (DHH) students and examined teachers' perceptions of students' engagement and comprehension processes.

**Methods:**

A mixed-methods design was conducted in Türkiye with 30 DHH middle school students enrolled in Grades 6 and 7. Students were assigned to guided reading, dialogic reading, or sign language instruction groups (*n* = 10 per group). The 8-week intervention used narrative texts and a common story-map framework addressing setting, time, characters, problem, solution, outcome, reaction, and main idea. Students' comprehension was assessed at baseline, weekly during instruction, immediately after the intervention, and 1 week later. Semi-structured interviews were conducted with six teachers.

**Results:**

All three groups demonstrated significant improvement over time, but the trajectories differed. Dialogic reading produced earlier and larger gains in higher-level narrative comprehension, particularly for problem, outcome, and main idea. At follow-up, both the dialogic reading and Sign Language Instruction groups outperformed the guided reading group on total comprehension (three-group omnibus test; ε^2^ = 0.75); the dialogic reading and Sign Language Instruction groups did not differ significantly from one another. Teachers described greater participation, elaborated responses, and increased attention to relationships among narrative events during dialogic reading.

**Conclusions:**

Accessible linguistic and visual supports may provide a common foundation for narrative comprehension, whereas dialogic interaction may offer additional opportunities for explanation, prediction, causal reasoning, and integration of narrative information.

## 1 Introduction

Reading comprehension is a central component of academic literacy because it requires readers not only to identify information in a text but also to integrate events, establish relationships, and construct a coherent representation of meaning. For many deaf and hard-of-hearing (DHH) students, developing these processes can be challenging, particularly when access to language and opportunities for sustained language interaction has been limited. Research has consistently documented variability in the academic and reading outcomes of DHH learners (Antia et al., 2009; Mayer et al., 2021) and has linked reading comprehension to language knowledge, vocabulary, syntactic and semantic processing, and inferential skills (Lederberg et al., 2013; Luckner and Handley, 2008; Marschark and Wauters, 2008; Marschark et al., 2015; Zhao et al., 2021). Recent systematic evidence further indicates that lexical knowledge is consistently associated with text-level reading comprehension in deaf readers and that competencies in sign language may also contribute to written-text comprehension through cross-modal linguistic processes (Mayer and Akamatsu, 2011; Olujić Tomazin et al., 2025). These findings underscore the need for literacy instruction that combines explicit comprehension support with accessible opportunities for language use.

In Türkiye, DHH students may receive education in specialized settings or in general education schools through inclusion/integration arrangements. Ministry of National Education regulations provide for special education schools for students with hearing loss, while inclusion/integration is also used as an educational arrangement across school levels (Ministry of National Education, 2021). These settings may differ in the extent to which students receive specialized instruction, communication support, and instructional adaptations. Such variation is relevant to literacy development because access to language and instructional support may influence students' opportunities to develop reading comprehension skills.

Research in Türkiye also indicates substantial variability in the reading development and achievement of DHH students. Karasu et al. (2012), using the Informal Reading Inventory with students in Grades 3–8, documented differences in reading performance between DHH and hearing students. Similarly, Genç et al. (2020) found that DHH students receiving inclusion education could identify many characters and main events in instructional-level texts but experienced difficulty with some story details and questions requiring the integration of information and prior experience. Turkish literature also emphasizes the relationship between language development and reading comprehension and the importance of early intervention for later literacy development (Özkan and Yücel, 2021).

Narrative comprehension provides an important context for examining these processes because understanding a story requires readers to organize information across multiple events and recognize relationships among characters, problems, actions, and outcomes. Story Grammar Theory conceptualizes narratives as structured representations in which components such as setting, characters, initiating events, problems, and outcomes are interconnected (Mandler and Johnson, 1977; Stein and Glenn, 1979). Story maps operationalize these relationships by making narrative structure visually explicit and may therefore provide useful support for learners who experience difficulties organizing connected text. Previous research has shown that story mapping and graphic organizers can facilitate narrative organization and comprehension, particularly when combined with explicit instruction and teacher support (Boulineau et al., 2004; Kim et al., 2004).

For DHH learners, however, narrative organization cannot be considered independently of linguistic accessibility. Reading comprehension depends on the interaction of linguistic knowledge and higher-order comprehension processes, including inference, integration, and the construction of coherent text representations (Holmer et al., 2016; Olujić Tomazin et al., 2025). Recent evidence indicates that both spoken-language and sign-language competencies can be relevant to text-level comprehension, highlighting the importance of providing accessible linguistic input rather than assuming a single modality of language access (Olujić Tomazin et al., 2025). Signed-language approaches to literacy instruction have consequently received increasing attention, with recent reviews identifying promising evidence while also emphasizing the limited and heterogeneous nature of the intervention literature (Dostal et al., 2025). Thus, an important instructional question is not simply whether DHH students receive accessible language, but how accessible language is used within instructional interactions to promote increasingly complex comprehension processes.

Three instructional approaches are particularly relevant to this question. Guided reading provides structured teacher support through activities such as prediction, vocabulary instruction, questioning, comprehension monitoring, and feedback. Its emphasis on gradual support and active participation may help learners develop strategic approaches to connected text (Schirmer and Schaffer, 2010a). Dialogic reading places greater emphasis on reciprocal interaction, prompting learners to recall, predict, explain, elaborate, and connect textual information through structured questioning and contingent feedback (Towson et al., 2016; Whitehurst et al., 1988, 1994). A recent three-level meta-analysis of 64 studies involving more than 10,000 typically developing children found moderate-to-large effects of dialogic reading on reading literacy and a large effect on reading comprehension, although the authors emphasized variation according to age and assessment characteristics (Tang et al., 2026). Importantly, however, this evidence base has focused predominantly on typically developing learners, leaving uncertainty regarding the applicability of these interactional mechanisms to DHH students. Sign Language Instruction, in contrast, prioritizes direct access to narrative content through sign language, visual representation, explicit vocabulary teaching, and teacher modeling. Recent evidence suggests that signed-language-based literacy interventions are promising but remain insufficiently studied across educational contexts (Dostal et al., 2025; Schirmer and Schaffer, 2010b).

Despite these developments, an important gap remains. Existing research has generally examined particular literacy practices separately, whereas few studies have directly compared guided reading, dialogic reading, and sign language instruction within the same instructional framework. Moreover, studies of DHH learners have often emphasized broad outcomes such as vocabulary, reading level, or overall comprehension, providing less information about how instructional approaches affect distinct components of narrative comprehension. This distinction is theoretically important because identifying explicitly stated elements such as characters or setting may place different demands on comprehension than integrating events to identify a problem, outcome, or main idea (Paris and Paris, 2003). The present study addresses this gap by using a common story-map framework across three instructional conditions and examining both the timing and magnitude of change across specific narrative components.

A further contribution is the integration of quantitative and qualitative evidence. Repeated quantitative assessments can identify whether and when students' comprehension changes, whereas teachers' accounts can provide contextual information about students' engagement, instructional interaction, and use of linguistic and visual supports. In the present mixed-methods study, qualitative data were therefore used to contextualize—not independently confirm—the quantitative findings. This approach allows the study to examine not only whether the instructional approaches were associated with changes in narrative comprehension, but also the classroom processes that may help explain differences in the observed trajectories.

Accordingly, the present study compared guided reading, dialogic reading, and sign language instruction for narrative comprehension among DHH students. Students were assessed across the intervention and at posttest and follow-up on eight narrative components: setting, time, characters, problem, solution, outcome, reaction, and main idea. Teachers' perceptions of students' engagement, narrative comprehension, instructional interaction, and linguistic and visual supports were also examined. By holding the narrative materials, story-map framework, instructional duration, and assessment procedures constant across conditions, the study sought to clarify whether differences in instructional interaction were associated with different patterns of narrative comprehension development. The purpose of this study was to compare the effects of guided reading, dialogic reading and direct instruction through sign language on the reading comprehension performance of DHH students. Specifically, the study examined students' performance on story map components, including setting, time, characters, problem, solution, outcome, reaction, and main idea, across pretest, instructional, posttest, and follow-up assessments. In addition, teachers' perceptions regarding the effectiveness and applicability of these instructional approaches were explored.

## 2 Method

### 2.1 Research design

This study employed an embedded mixed-methods intervention design to examine the effects of three reading instructional approaches—guided reading, dialogic reading, and direct instruction through sign language instruction on the narrative comprehension of deaf and hard-of-hearing (DHH) students. The quantitative component was used to examine changes in students' reading comprehension across instructional conditions and repeated assessment points, whereas the qualitative component involved semi-structured interviews with teachers to explore their perceptions of students' engagement, comprehension processes, and the applicability of the instructional approaches. The qualitative findings were used to contextualize and help interpret the quantitative results, consistent with mixed-methods approaches that integrate quantitative and qualitative evidence within a single study (Creswell and Plano Clark, 2018; Creswell and Hirose, 2019).

### 2.2 Participants

#### 2.2.1 Deaf and hard of hearing (DHH) students

Thirty deaf and hard-of-hearing (DHH) students enrolled in Grades 6 and 7 at a public school serving students with hearing loss participated in the study. The middle-school level was selected because it represents a transition from learning to read toward reading to learn, as students increasingly use reading comprehension to access more complex academic content. This transition is particularly important for DHH students' academic participation. Eligibility criteria were: (a) no diagnosed disability other than hearing loss; (b) severe-to-profound hearing loss; (c) ability to read a simple narrative text independently; (d) ability to respond using Turkish Sign Language (TID), spoken/written Turkish, or both; and (e) performance at the instructional reading level on the FOOE preliminary assessment, corresponding to a score within the instructional-level range (59–89).

The preliminary screening was conducted using the Turkish Informal Reading Inventory (FOOE). A total of 38 students were initially assessed. Students were eligible for participation if they demonstrated instructional-level reading comprehension on the FOOE and met all other predefined inclusion criteria. Following this screening process, 30 students met the eligibility criteria and were included in the study. Thus, the final sample consisted of 30 DHH students, with 10 students assigned to each of the three instructional conditions. Although the participants were enrolled in Grades 6 and 7, the FOOE was used to determine their instructional reading level for study eligibility rather than to assign them to a grade-level reading category. The demographic and audiological characteristics of the study group are presented in Table 1.

**Table 1:** Demographic and audiological characteristics of the study group.

Variable	Category	f	%
Gender	Girl	18	60.0
	Boy	12	40.0
Grade level	6^th^ grade	13	43.3
	7^th^ grade	17	56.7
Age	11 years	2	6.7
	12 years	11	36.7
	13 years	12	40.0
	14 years	2	6.7
	15 years	3	10.0
Communication preference	Sign Language Only (SL)	20	66.7
	Sign and Spoken Language (SL + SpL)	10	33.3
Hearing loss in family	Yes	21	70.0
	No	9	30.0
Hearing device status	Non-user	11	36.7
	Hearing aid (Unilateral)	8	26.7
	Hearing sid (Bilateral)	2	6.7
	Cochlear implant	9	30.0
Degree of Hearing Loss (dB)	71–80 dB	6	20.0
	81–90 dB	17	56.7
	91 dB and above	7	23.3

An examination of the demographic data presented in Table 1 shows that the majority of participants were girls (f = 18, 60.0%). Two-thirds of the students (66.7%) were found to prefer Sign Language (SL) only as their communication method. In addition, a high proportion of students (f = 21, 70.0%) were found to have a family history of hearing loss. Regarding hearing device use, the largest group in the sample did not use a hearing device (f = 11, 36.7%), and more than half of the participants (56.7%) had a hearing loss in the 81-90 dB range.

As previously stated, descriptive statistics based on the scores obtained from the FOOE preliminary assessment were calculated and are presented in Table 2, to determine whether participants met the inclusion criterion and to establish the baseline equivalence of the groups prior to the intervention.

**Table 2:** Descriptive statistics for the FOOE preliminary assessment.

Instructional condition	Minimum	Maximum	Mean
Guided reading	69	75	72.1
Dialogic reading	66	75	71.3
Sign language instruction	66	77	72.4

As previously stated, descriptive statistics based on the scores obtained from the FOOE preliminary assessment were calculated and are presented in Table 2 to document participants' instructional reading levels and to examine the baseline equivalence of the three intervention groups prior to the intervention. The minimum scores obtained by the three groups were 69 for Guided Reading, 66 for Dialogic Reading, and 66 for Sign Language Instruction, while the corresponding maximum scores were 75, 75, and 77, respectively. All participants therefore obtained scores within the instructional-level range specified in the FOOE framework (59–89). These findings indicate that all students demonstrated instructional-level reading comprehension on the preliminary assessment and met the study's eligibility criterion. The group means were 72.1 for Guided Reading, 71.3 for Dialogic Reading, and 72.4 for Sign Language Instruction, indicating broadly comparable performance across the three groups at baseline.

#### 2.2.2 Teacher participants

Six teachers who were actively involved in teaching the participating students contributed to the qualitative component. The group included two teachers of DHH students, two special education teachers, and two Turkish language teachers. Their professional experience ranged from 1 to more than 31 years, and their experience in the participating school ranged from 1 to more than 11 years. Three teachers reported good proficiency in Turkish Sign Language, one reported intermediate proficiency, and two reported no sign-language proficiency (see Table 3). The six teachers did not deliver the intervention or receive intervention-specific training. They observed the instructional sessions and, following the intervention, participated in interviews about the students' progress and reading comprehension based on their knowledge of the students.

**Table 3:** Characteristics of the teachers in the qualitative interview study group.

Variable	Category	*f*	%
Gender	Female	2	33.3
	Male	4	66.7
Teaching specialty	Teacher of the deaf and hard of hearing	2	33.3
	Special education teacher	2	33.3
	Turkish language teacher	2	33.3
Age group	20-30 Years	1	16.7
	41-50 Years	3	50.0
	51 Years and Above	2	33.3
Professional experience	1-10 Years	1	16.7
	11-20 Years	2	33.3
	21-30 Years	1	16.7
	31 Years and above	2	33.3
Experience in current school	1-5 Years	2	33.3
	6-10 Years	3	50.0
	11 Years and above	1	16.7
Sign language proficiency	Good	3	50.0
	Intermediate	1	16.7
	None	2	33.3

An examination of Table 3, which presents the teacher group for the qualitative data collection, shows that two teachers from each of the Teacher of the Deaf and Hard of Hearing, Special Education, and Turkish Language branches (f = 2, 33.3% each) were equally represented in the interview group. The participating teachers' professional experience spanned a fairly wide range, with half of the group (50.0%) having 21 years or more of professional experience. Regarding sign language proficiency, the two teachers from the Turkish Language branch (f = 2, 33.3%) were found not to know sign language. This is expected to reflect different branch-based perspectives on students' language and comprehension skills in the qualitative interviews.

### 2.3 Data collection instruments

#### 2.3.1 Demographic and audiological information form

A researcher-developed demographic and audiological information form was used to collect information on students' age, sex, grade level, degree of hearing loss, communication modality, family history of hearing loss, and hearing-device use.

#### 2.3.2 Informal reading inventory (FOOE)

The Turkish Informal Reading Inventory (*Formel Olmayan Okuma Envanteri* [FOOE]) was used to assess students' reading and reading-comprehension skills and to determine their eligibility for participation in the study. The FOOE is an informal assessment tool designed to evaluate reading performance across different instructional levels (Karasu et al., 2013). Previous research has also demonstrated the use of the FOOE in assessing the reading skills of students with hearing loss, supporting its relevance to the present study (Karasu et al., 2012). In the present study, the Level 1 narrative text *Pazar*, consisting of 65 words and 20 sentences, was administered. The text assesses comprehension across three dimensions: characters (25 points), main events (50 points), and details (25 points), yielding a total score of 100. Students read the text and subsequently retold it using spoken Turkish, Turkish Sign Language (TID), or both, according to their preferred communication modality. The FOOE was used for participant screening and baseline characterization rather than as the primary outcome measure. Although the participants were enrolled in Grades 6 and 7, the use of the Level 1 FOOE text was intended specifically to determine their instructional reading level for study eligibility. It was not used to characterize the participants as first-grade readers or to establish a grade-equivalent reading level. In contrast, the approximately 100-word narrative texts described below were used as standardized materials across participants. The same approximate text length was maintained to provide consistency and comparability in the assessment and instructional procedures. Thus, the FOOE and the narrative texts served distinct purposes: the FOOE was used to identify participants' instructional reading level, whereas the approximately 100-word narrative texts provided a standardized basis for assessing and supporting narrative reading comprehension.

#### 2.3.3 Narrative texts

Eleven narrative texts selected from the story set developed by Cora-Ince (2007) were used. The texts were reviewed and adapted based on expert judgment to ensure linguistic accessibility, consistency in narrative structure, and suitability for the participants' instructional reading level. Each text was approximately 100 words long, written in the third person, and avoided figurative expressions. All narratives included the same eight target components: setting, time, characters, problem, solution, outcome, reaction, and main idea. Visual illustrations accompanying each story were provided to support access to the narrative content. Eight stories were used during instruction, whereas two were used for pretest/posttest assessment and one was used for follow-up assessment. Assessment texts differed from those used during instruction to reduce the possibility that performance reflected memorization of previously taught stories.

#### 2.3.4 Story map and monitoring form

A story-map framework adapted from Boulineau et al. (2004) was used to support students' identification and organization of narrative information. The map included eight components: setting, time, characters, problem, solution, outcome, reaction, and main idea. Visual symbols were added to the components to increase accessibility for DHH students. To monitor students' performance during instruction, a researcher-developed monitoring form was used. Each story component was scored on a three-level scale: 0 = incorrect or incomplete response, 1 = response completed with assistance, and 2 = correct, complete, and independent response. The form was used to document students' progress across instructional sessions. Weekly scores were collected during instruction and therefore represent progress-monitoring data rather than an independent outcome measure. Because the type and amount of assistance that could earn a score of 1 varied by design across the three conditions (e.g., dialogic prompting vs. direct modeling), weekly comparisons should be interpreted as indexing instructional progress under condition-specific support rather than unprompted performance. In contrast, the posttest and follow-up assessments were administered without instructional prompting, so that scores of 1 (partial/assisted response) at posttest and follow-up reflect only the minimal support built into the standard testing procedure, not condition-specific instructional assistance.

#### 2.3.5 Qualitative data collection and analysis

Interviews were conducted individually with each of the six teachers in Turkish, lasted approximately 1 h, and were audio recorded and transcribed verbatim. The data were analyzed inductively using thematic analysis following Braun and Clarke (2006). The transcripts were independently reviewed and coded by the researcher and a field expert. Inter-coder agreement was 95%. Coding discrepancies were discussed and resolved through consensus. Two rounds of coding were conducted, and codes and emerging themes were reviewed and refined iteratively.

#### 2.3.6 Teacher interview form

A semi-structured teacher interview form developed by the researchers was used to collect qualitative data. The interview questions addressed teachers' perceptions of the instructional approaches, their perceived contribution to students' reading and reading-comprehension skills, and their observations of the instructional process. Demographic and professional information, including teaching specialization, age, gender, years of professional experience, years of service at the participating school, and Turkish Sign Language proficiency, was also collected.

### 2.4 Research setting

The study was conducted at a public school serving DHH students. Before data collection, the researchers met with the school administration, participating teachers, and relevant school personnel to explain the purpose, scope, and procedures of the study. Institutional and ethical approvals were obtained before the study commenced, and the instructional schedule was coordinated with the school administration. During the 1 week, students were screened for eligibility using the FOOE. Students who met the predefined participation criteria were subsequently included in the pretest assessment. Pretest assessments were administered individually and were completed over approximately 1 week.

Instructional sessions were conducted in an available classroom within the school. The classroom was equipped with an interactive whiteboard, desks, chairs, and a computer. Instruction was delivered in small groups of two students during scheduled Turkish language lessons. Following completion of the pretest, the 8-week instructional intervention was implemented 3 days per week, with each session lasting approximately 80 min (two 40-min class periods). All instructional conditions were implemented using a small-group format.

Posttest assessments were administered individually following completion of the intervention and were completed over approximately 1 week. A follow-up assessment was conducted 2 week after the posttest to examine the maintenance of students' performance. Across screening, pretest, intervention, posttest, and follow-up procedures, the study was completed over approximately 12 weeks. Screening and assessment procedures were conducted individually, whereas instructional sessions were conducted in small groups.

### 2.5 Procedure

Before the intervention, 38 students were screened using the FOOE to determine eligibility. Students who demonstrated instructional-level reading comprehension and met the other predefined inclusion criteria were eligible to participate. Following the screening and eligibility assessment, 30 students were included in the study. These 30 students were subsequently assigned to one of three instructional conditions: guided Reading (*n* = 10), Dialogic Reading (*n* = 10), or Direct Instruction Through Sign Language (*n* = 10). All 30 participants completed the 8-week intervention and the subsequent posttest and follow-up assessments. No participants withdrew from the study or were lost to follow-up; therefore, the final analyzed sample consisted of 30 students.

Across conditions, the same narrative materials, visual supports, story maps, instructional schedule, and assessment procedures were used. The same interventionist and sign-language specialist delivered the instructional procedures across all three conditions. The classroom teachers did not deliver the intervention but observed the sessions and subsequently participated in the qualitative interviews. The instructional condition was the principal difference between groups. The instructional procedures are summarized in Table 4.

**Table 4:** Comparison of instructional procedures across the three reading conditions.

Instructional component	Guided reading	Dialogic reading	Direct instruction through sign language
Activation of prior knowledge	Teacher-guided questions	Open-ended questions and discussion	Structured elicitation of prior knowledge
Preview of title and visuals	Prediction based on title and illustrations	Prediction and inference based on title and illustrations	Introduction of characters and events
Vocabulary instruction	Explanation of key vocabulary	Discussion and contextual elaboration of key concepts	Explicit instruction in key vocabulary
Text presentation	Supported student reading	Dialogic reading with frequent dialogue	Sentence-by-sentence reading with TID interpretation
During-reading support	Prompts, guidance, and comprehension checks	PEER- and CROWD-based questioning	Modeling, explanation, and comprehension checks
Narrative-component instruction	Guided identification of story components	Collaborative identification through discussion	Explicit identification and modeling of story components
Story-map completion	Guided completion	Discussion-based collaborative completion	Independent completion following explicit instruction
Feedback	Prompts and corrective feedback	Response expansion, reformulation, and feedback	Immediate corrective feedback
Reinforcement	Verbal praise and selected rewards	Verbal praise and selected rewards	Verbal praise and selected rewards
Communication support	TID support as needed	TID support as needed	TID used as the primary instructional modality

All three conditions used the same narrative texts, visual materials, story maps, instructional schedule, and reinforcement procedures. The primary distinction among conditions concerned the instructional interaction and language-support procedures used to facilitate comprehension. Instruction was delivered over 8 weeks, three sessions per week, for a total of 24 instructional sessions.

### 2.6 Data analysis

The alpha level for all inferential tests was set at 0.05. Because the outcome scores were ordinal (0/1/2 per story component) and group sizes were small (*n* = 10 per condition), between-group comparisons at each measurement occasion were conducted using the Kruskal-Wallis H test, and within-group change across the eleven measurement occasions (pretest, Weeks 1–8, posttest, and follow-up) was examined using the Friedman test. Effect sizes for both tests are reported as epsilon-squared (ε^2^), interpreted following Tomczak and Tomczak-Łukaszewska (2014); Cohen (1988). Where an omnibus test was significant, pairwise *post hoc* comparisons (Dunn's test for Kruskal-Wallis; Durbin/Conover-type tests for Friedman) were conducted with a Bonferroni correction applied within each family of comparisons to control the family-wise error rate. The total narrative comprehension score at follow-up was pre-specified as the primary outcome and the three-group comparison as the primary contrast; analyses of the eight individual story components at each measurement occasion (Supplementary Tables 1–21) are reported as exploratory and are interpreted accordingly. All analyses were conducted in IBM SPSS 27. There were no missing data; tied ranks were handled using the standard mid-rank correction implemented in the software's non-parametric procedures. Analysis of teacher interview data is described in the Qualitative Findings section below.

### 2.7 Instructional conditions

Each instructional week focused on one approximately 100-word narrative text. The same text was used across the three 80-min sessions of that week, allowing students to engage with the narrative through the respective instructional approach across multiple sessions. The eight instructional texts were therefore implemented sequentially across the 8-week intervention.

#### 2.7.1 Guided reading

Guided reading sessions incorporated pre-reading, during-reading, and post-reading activities designed to support students' comprehension through structured teacher guidance. For example, during instruction with the *Respect for Older Adults* story, the teacher activated students' prior knowledge using questions such as “Have you ever ridden a bus?,” “Where have you traveled by bus?,” and “To whom should we offer our seat on a bus?” Questions were presented using spoken language and Turkish Sign Language (TID). A teacher proficient in TID provided communication support throughout the sessions, while the interventionist, sign-language specialist, and classroom teacher worked collaboratively to facilitate students' participation.

Before reading, students examined the title and illustrations and were asked to predict the topic of the story. Characters, events, and key concepts were introduced using visual materials displayed on a computer and interactive whiteboard. Key vocabulary, including *help, love, compassion*, and *respect*, was presented with corresponding visual representations. Students then took turns reading the story. The teacher provided vocabulary explanations and prompts when needed to support text monitoring and comprehension. During reading, questions focused on narrative components such as setting, time, characters, problem, and solution.

After reading, students retold the story and completed the eight components of the story map: setting, time, characters, problem, solution, outcome, reaction, and main idea. The teacher provided prompts when necessary to support students in identifying appropriate information. At the end of each session, students received verbal praise and, where appropriate, small preferred rewards.

#### 2.7.2 Dialogic reading

Dialogic reading sessions were designed to promote active student participation, verbal and signed expression, and meaning construction through structured interaction around the text. During the *Respect for Older Adults* story, the teacher first used the title and illustrations to focus students' attention and posed prediction questions such as “What do you think this story is about?” and “What might Fatih have done?”

Before reading, key concepts such as *help, love, compassion*, and *respect* were discussed in greater depth. These concepts were presented with visual materials and linked to students' everyday experiences, with TID support provided as needed. Characters and major events were also presented visually to support students' construction of a coherent representation of the story. Compared with the other conditions, visual supports and key-vocabulary discussions were used more intensively in the dialogic reading condition.

During reading, the teacher paused frequently to ask open-ended questions and encourage students to elaborate on their responses. The dialogic reading procedures incorporated the PEER (Prompt, Evaluate, Expand, Repeat) and CROWD (Completion, Recall, Open-ended, Wh- questions, and Distancing) strategies described by Whitehurst et al. (1994). Students were encouraged to recall events, complete partially presented statements, answer open-ended questions, explain causal relationships, and relate story events to their own experiences.

Students' responses were evaluated and, when appropriate, expanded or reformulated by the teacher. Students were then encouraged to repeat or elaborate on the expanded responses. The questioning sequence was systematically linked to the eight story-map components. For example, questions such as “What did Fatih see on the bus?” targeted recall of story events; “Why did Fatih give his seat to the older woman?” targeted causal reasoning; and “Have you ever helped an older person?” and “What would you have done?” encouraged connections between the story and students' own experiences.

Questions were presented through spoken language and TID, and students were encouraged to express their ideas in sufficient detail. Following the reading, students discussed the story, evaluated characters' actions, and attempted to explain its main idea before completing the story map. The interventionist, sign-language specialist, and classroom teacher worked collaboratively throughout the sessions to facilitate participation and comprehension.

Examples of the PEER sequence and CROWD questioning strategies used during the dialogic reading sessions are provided in Appendix A.

#### 2.7.3 Direct instruction through sign language

The direct instruction condition used a systematic and highly structured approach in which Turkish Sign Language served as the primary modality for supporting access to the narrative content. During instruction with the *Respect for Older Adults* story, students' prior knowledge was activated through questions such as “To whom should we offer our seat on a bus?” and “How can we help older people?” These questions were presented in TID, and students were encouraged to share relevant prior experiences. Characters, events, and key concepts were subsequently introduced using visual materials displayed on a computer and interactive whiteboard. Key vocabulary, including *help, love, compassion*, and *respect*, was explicitly introduced through visual representations and TID explanations.

The story was then presented sentence by sentence. The teacher read each sentence and provided its meaning in TID before proceeding to the next sentence. Important vocabulary and events were explicitly explained, and students were asked to reproduce key information through TID. The interventionist, sign-language specialist, and classroom teacher coordinated their roles throughout the sessions to maintain students' attention and facilitate access to the narrative content.

Following presentation of the complete story, the teacher explicitly introduced the eight narrative components—setting, time, characters, problem, solution, outcome, reaction, and main idea—and modeled how each component could be identified and represented on the story map. Students subsequently identified the same components independently and completed the story map. Immediate corrective feedback was provided when students produced inaccurate or incomplete responses, and correct responses were reinforced at the end of each session.

## 3 Results

### 3.1 Preliminary statistics

The reading comprehension levels of the hearing-impaired students participating in the study (n = 30) were examined prior to the interventions through a preliminary assessment. The results of the Kruskal-Wallis H test conducted to compare the groups' baseline levels showed no statistically significant difference among the groups [H(2) ranging from 0 to 0.92, all *p* > 0.05] (see Supplementary Table 1). This finding confirms that the students were equivalent in terms of reading comprehension skills at the outset of the study. Therefore, any differences observed between the groups after the 8-week instructional process can be attributed to the effects of the intervention methods applied (Dialogic reading, guided reading, and sign language instruction).

#### 3.1.1 The 8-week intervention period and weekly progress

The weekly measurements taken over the 8-week intervention period yielded consistent findings regarding students' progress. The total weekly reading comprehension scores obtained according to instructional method during the intervention period are presented in Table 5. In addition, detailed weekly statistical comparisons for each of the eight story components (setting, time, character, problem, solution, outcome, reaction, and main idea) during the intervention are provided in the Supplementary Tables 2–9 section. Within-group longitudinal progress across the measurement points was analyzed using the Friedman test. For all pairwise *post-hoc* comparisons, the Bonferroni correction was rigorously applied to control the family-wise error rate across multiple tests, and adjusted *p*-values (*p*_*adj*_) are reported to protect against Type I errors resulting from multiple testing.

**Table 5:** Total reading comprehension scores by instructional method during the 8-week intervention period.

Week	Guided reading (*n* = 10) Mrank | M±SD | Med (IQR)	Dialogic reading (*n* = 10) Mrank | M±SD | Med (IQR)	Sign language instruction (*n* = 10) Mrank | M±SD | Med (IQR)	H (df = 2)	*p*	ε2
1	9.95 | 2.50 ± 0.85 | 2.00 (1.25)	17.65 | 3.70 ± 1.49 | 3.50 (1.50)	18.90 | 3.90 ± 1.52 | 4.00 (2.25)	6.57	0.038^*^	0.17
2	7.85 | 3.50 ± 1.96 | 3.00 (0.00)	19.30 | 6.80 ± 2.86 | 6.50 (6.00)	19.35 | 6.80 ± 2.10 | 7.00 (3.25)	11.81	0.003^**^	0.36
3	7.65 | 6.40 ± 0.70 | 6.50 (1.00)	20.90 | 10.30 ± 2.71 | 11.50 (6.00)	17.95 | 9.30 ± 2.41 | 9.00 (3.75)	12.9	0.002^**^	0.4
4	6.30 | 8.80 ± 0.42 | 9.00 (0.25)	23.65 | 13.40 ± 2.07 | 14.00 (4.00)	16.55 | 10.90 ± 1.20 | 11.00 (0.75)	20.82	0.000^***^	0.7
5	8.05 | 9.60 ± 1.17 | 9.50 (2.25)	23.85 | 14.20 ± 1.99 | 15.00 (3.25)	14.60 | 11.20 ± 1.48 | 11.00 (3.00)	16.58	0.000^***^	0.54
6	8.40 | 10.60 ± 1.58 | 11.00 (2.50)	25.30 | 15.00 ± 1.05 | 15.00 (2.00)	12.80 | 11.70 ± 1.42 | 12.00 (2.25)	20.22	0.000^***^	0.67
7	11.50 | 12.90 ± 1.60 | 13.50 (2.00)	25.05 | 15.70 ± 0.68 | 16.00 (0.25)	9.95 | 12.80 ± 1.32 | 13.00 (1.25)	18.67	0.000^***^	0.62
8	7.55 | 13.70 ± 1.06 | 14.00 (1.50)	23.60 | 15.80 ± 0.42 | 16.00 (0.25)	15.35 | 14.90 ± 0.74 | 15.00 (1.25)	18.12	0.000^***^	0.6

Mrank = Mean Rank. H = Kruskal-Wallis H statistic. ε^2^= Effect size. ^*^p < 0.05, ^**^p < 0.01, ^***^p < 0.001. Effect Size Interpretation: ε^2^ < 0.01 = Very Small, 0.01-0.06 = Small, 0.06-0.14 = Medium, > 0.14 = Large (Tomczak and Tomczak-Łukaszewska, 2014).

An examination of Table 5 indicates statistically significant differences in reading comprehension scores among the three instructional groups across all eight intervention weeks (*p* < 0.05). In the initial adaptation phase (Week 1), between-group differences were modest but significant (*H*(2) = 6.57, *p* = 0.038, ε^2^ = 0.17), with Sign Language Instruction (*Mrank* = 18.90, *M* = 3.90 ± 1.52, *Mdn* = 4.00) and Dialogic Reading (*Mrank* = 17.65, *M* = 3.70 ± 1.49, *Mdn* = 3.50) demonstrating higher baseline acquisition than Guided Reading (*Mrank* = 9.95, *M* = 2.50 ± 0.85, *Mdn* = 2.00).

Between Weeks 2 and 4, the superiority of Dialogic Reading became increasingly pronounced, with effect sizes shifting from large to exceptionally strong (ε^2^ rising from 0.36 to 0.70). By Week 4, Dialogic Reading achieved a substantially higher mean rank (*Mrank* = 23.65, *M* = 13.40 ± 2.07, *Mdn* = 14.00, *IQR* = 4.00) relative to Sign Language Instruction (*Mrank* = 16.55, *M* = 10.90 ± 1.20, *Mdn* = 11.00, *IQR* = 0.75) and Guided Reading (*Mrank* = 6.30, *M* = 8.80 ± 0.42, *Mdn* = 9.00, *IQR* = 0.25;*H*(2) = 20.82, *p* < 0.001). During the consolidation phase spanning Weeks 5 through 7, Dialogic Reading sustained its clear advantage over both alternatives (ε^2^ ranging from 0.54 to 0.67). The final intervention session (Week 8) confirmed the stability of this pattern, wherein Dialogic Reading maintained peak performance (*Mrank* = 23.60, *M* = 15.80 ± 0.42, *Mdn* = 16.00) compared to Sign Language Instruction (*Mrank* = 15.35, *M* = 14.90 ± 0.74, *Mdn* = 15.00) and Guided Reading (*Mrank* = 7.55, *M* = 13.70 ± 1.06, *Mdn* = 14.00;*H*(2) = 18.12, *p* < 0.001, ε^2^ = 0.60).

To complement the between-group findings (Supplementary Table 21), pairwise comparisons examining longitudinal progress across measurement points revealed significant trajectories over time. When evaluating unadjusted significance levels, meaningful progress relative to the pre-test (PrTOP) was observable as early as Week 3 (*z* = 2.259, *p* = 0.024). Under the conservative Bonferroni correction (*p*_*adj*_ < 0.05), cumulative gains became statistically robust from Week 6 onward (PTOP–TOP6: *z* = 3.674, *p*_*adj*_ = 0.013) and remained significant through subsequent intervention weeks, *post-test* (STOP, *p*_*adj*_ < 0.001), and follow-up assessments (FTOP, *p*_*adj*_ < 0.001). Furthermore, early intervention sessions (TOP1 and TOP2) differed significantly from the late and final assessments even after multi-comparison adjustment (e.g., TOP1–TOP7: *z* = −4.079, *p*_*adj*_ = 0.002; TOP1–STOP: *z* = −5.731, *p*_*adj*_ < 0.001; TOP2–STOP: *z* = −4.922, *p*_*adj*_ < 0.001). Importantly, comparisons between the final intervention week (TOP8), the post-test (PoTOP), and the follow-up measurement (ITOP) yielded non-significant differences (*p* > 0.05, *p*_*adj*_ = 1.000), demonstrating a performance ceiling in the final stages and evidencing that comprehension gains were successfully maintained beyond the intervention period. To visually illustrate these developmental patterns across the intervention timeline, weekly mean total comprehension trajectories for each instructional condition are displayed in Figure 1.

**Figure 1:**
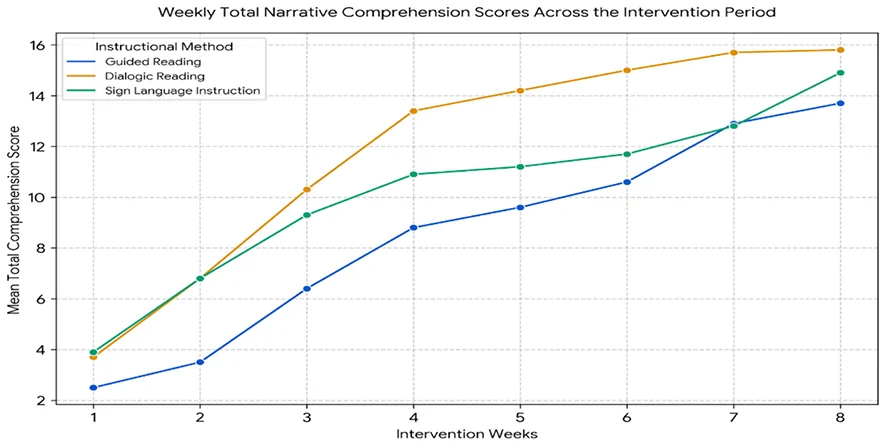
Weekly mean total narrative comprehension scores across the 8-week intervention period by instructional condition.

#### 3.1.2 Differential effects on story components

When reading comprehension achievement was analyzed across the different structural and cognitive components of the story, clear differences emerged in the effectiveness of the methods (for detailed results, see Supplementary Tables 2–9).

In this study, story comprehension skills were examined at three levels. The first level, basic structural information (setting and character), corresponds to the “setting” component of story grammar, which introduces the main character (s) and describes the social, physical, or temporal context of the story (Stein and Glenn, 1979). The second level, intermediate-level comprehension (problem–solution– outcome), reflects the causal chain of events that defines an episode, beginning with an initiating event and ending in a outcome (Stein and Glenn, 1979). The third level, advanced-level comprehension (main idea), corresponds to what Paris and Paris (2003) classify as an implicit narrative element requiring the integration of multiple story events into a coherent, narrative-level theme, as opposed to explicit elements such as character, setting, and problem, which can be identified directly from the story (Paris and Paris, 2003).

In the “basic structural information” category (setting, time, character), which requires only surface-level processing and is directly stated in the text, no consistent or significant difference was observed between the groups over the 8 weeks (mostly *p* > 0.05). This suggests that DHH were able to grasp the directly stated basic elements of the story to a similar extent regardless of instructional method.

In contrast, in the “intermediate-level comprehension” dimension (problem, solution, and outcome), which requires establishing cause-and-effect relationships, the advantage of dialogic reading becomes apparent. Beginning in Week 4, the dialogic reading group achieved significantly higher success in problem identification, and from Week 6 onward it diverged clearly from the other two groups in the ability to make sense of the story's outcome processes [H (2) = 18.35, *p* < 0.001, ε^2^ = 0.64].

When “advanced-level comprehension” (main idea extraction), one of the highest-order cognitive skills, was examined, the dialogic reading group was found to display the strongest and most consistent performance from the 1 week to the last (*p* < 0.001, ε^2^ ranging from 0.70 to 1.00). This finding suggests that dialogic interaction may support students' ability to integrate the text as a whole, although the present design does not permit conclusions about which specific features of the dialogic reading package were responsible for this pattern.

#### 3.1.3 Posttest and follow-up assessment: retention

The overall results of the posttest and follow-up measurements, administered to assess whether intervention effects were maintained 1 week after the end of instruction (short-term retention), are presented in Table 6, and detailed component-based breakdowns are provided in the Supplementary Tables 10–12.

**Table 6:** Between-group comparisons in posttest and follow-up (retention) assessments.

Measurement/component	Guided reading (*n* = 10) Mrank | M±SD | Med (IQR)	Dialogic reading (n = 10) Mrank | M±SD | Med (IQR)	Sign language instruction (*n* = 10) Mrank | M±SD | Med (IQR)	H (df = 2)	*p*	ε2	Significant difference (Bon ferroni)
Posttest - Problem	7.50 | 1.10 ± 0.32 | 1.00 (0.00)	21.00 | 2.00 ± 0.00 | 2.00 (0.00)	18.00 | 1.80 ± 0.42 | 2.00 (0.25)	18.59	0.000^***^	0.65	DR > GR^***^ SLI > GR^**^
Posttest - Main Idea	5.50 | 1.00 ± 0.00 | 1.00 (0.00)	20.50 | 2.00 ± 0.00 | 2.00 (0.00)	20.50 | 2.00 ± 0.00 | 2.00 (0.00)	29	0.000^***^	1	DR > GR^***^ SLI > GR^***^
Follow-up - Problem	8.00 | 1.00 ± 0.00 | 1.00 (0.00)	21.50 | 1.90 ± 0.32 | 2.00 (0.00)	17.00 | 1.60 ± 0.52 | 2.00 (1.00)	16.24	0.000^***^	0.56	DR > GR^***^ SLI > GR^*^
Follow-up - Reaction	6.50 | 1.10 ± 0.32 | 1.00 (0.00)	20.00 | 2.00 ± 0.00 | 2.00 (0.00)	20.00 | 2.00 ± 0.00 | 2.00 (0.00)	24.86	0.000^***^	0.88	DR > GR^***^ SLI > GR^***^
Follow-up - Main Idea	7.00 | 1.00 ± 0.00 | 1.00 (0.00)	22.00 | 2.00 ± 0.00 | 2.00 (0.00)	17.50 | 1.70 ± 0.48 | 2.00 (1.00)	20.73	0.000^***^	0.73	DR > GR^***^ SLI > GR^**^
Follow-up - Total	7.00 | 13.00 ± 0.00 | 13.00 (0.00)	24.25 | 15.90 ± 0.32 | 16.00 (0.00)	15.25 | 14.40 ± 1.26 | 14.50 (1.50)	21.37	0.000^***^	0.75	DR > GR^***^ SLI > GR^*^

DR, Dialogic Reading; GR, Guided Reading; SLI, Sign Language Instruction. Mrank, Mean Rank. *M* = Mean. *SD* = Standard Deviation. Med = Median. IQR = Interquartile Range (*Q*_3_ − *Q*_1_). *H* = Kruskal-Wallis *H* statistic. ε^2^ = Effect size. ^***^p < 0.001, ^**^p < 0.01, ^*^p < 0.05. Effect Size Interpretation: ε^2^ < 0.01 = Very Small, 0.01 − 0.06 = Small, 0.06 − 0.14 = Medium, >0.14 = Large (Tomczak and Tomczak-Łukaszewska, 2014).

All three groups showed statistically significant within-group temporal changes (Friedman test, all p < 0.001; see Supplementary Table 12). Dialogic Reading demonstrated the largest and most consistent effect sizes (ε^2^ = 0.59 on average), followed by Sign Language Instruction (ε^2^ = 0.45), and Guided Reading (ε^2^ = 0.34). Notably, the temporal development in Dialogic Reading began earlier (Week 4) compared to other groups, suggesting faster and more sustained improvements.

An examination of the posttest findings in Table 6 shows that dialogic reading produced a substantial effect, particularly in main idea extraction. Notably, the epsilon-squared (ε^2^) value of 1.00 for the main idea component indicates complete rank separation driven by a pronounced ceiling effect, as students in both the Dialogic Reading and Sign Language Instruction conditions consistently reached the maximum possible scores.

The follow-up assessments conducted 1 week after the intervention also show that the differences between groups persisted, with the reaction component (comprehension of characters' reactions to events) reaching a very large effect size (ε^2^= 0.88). In the overall total scores of the follow-up measurement, both the Dialogic Reading and Sign Language Instruction groups significantly outperformed the Guided Reading group (ε^2^ = 0.75). While Dialogic Reading maintained the highest relative mean rank, these results demonstrate the effectiveness of both dialogic interaction and direct sign language scaffolding in consolidating learning for short-term retention.

For each story component, progress across the preliminary assessment, the 8-week process, and the posttest and follow-up measurements was tested using the Friedman test. An examination of the rates of progress shows that all three methods achieved progress over time in basic skills, although the points at which this progress accelerated differed considerably. Detailed tables showing the Friedman test results and the significant differences over time (*post hoc*) for each component are provided in the Supplementary Tables 13–20.

Although significant progress over time was observed across all methods, progress in the setting and time components began earlier in the *dialogic reading group* (Weeks 3 and 5) than in the other methods. In the character component, all methods followed a fairly similar course of progress.

In the dialogic reading group, problem identification and solution-finding skills showed a rapid jump in Weeks 4 and 5 (ε^2^ = 0.81), whereas this progress was delayed until Weeks 6 and 7 in the *guided reading* and *sign language instruction* groups, respectively.

In “main idea” extraction, considered the peak of the text comprehension process, *the guided reading* and *sign language instruction* groups achieved significant progress only toward the very end of the intervention (at the posttest and follow-up measurements). In contrast, the dialogic reading group showed a systematic, consistent, and strong increase in main idea extraction from Week 5 onward (ε^2^= 0.81).

### 3.2 Qualitative findings: teachers' perspectives

The qualitative findings were examined to complement and contextualize the quantitative results by exploring teachers' observations of students' engagement, narrative comprehension, instructional interaction, and access to linguistic and visual supports. The analysis yielded four closely related themes: (a) active engagement in comprehension, (b) development of narrative comprehension, (c) interaction and instructional scaffolding, and (d) accessible linguistic and visual support. Each theme included two subthemes, as summarized in Table 7. Teachers' observations broadly converged with the quantitative findings, particularly with respect to the progressive improvement observed across all three instructional conditions and the comparatively stronger performance of the dialogic reading group on higher-level comprehension components.

**Table 7:** Summary of teachers' perceptions and their relationship to quantitative findings.

Theme	Descriptive qualitative finding	Relationship to quantitative findings
Active engagement in comprehension	Teachers observed increased attention, participation, and willingness to respond, particularly during dialogic reading activities.	Consistent with the stronger and earlier improvement observed in the dialogic reading condition.
Development of narrative comprehension	Teachers reported improved identification and organization of narrative elements, including problem–solution relationships and the main idea.	Converges with the stronger gains observed for problem, outcome, and main-idea comprehension in the dialogic reading condition.
Interaction and instructional scaffolding	Teachers perceived questioning, prompting, feedback, and discussion as supporting explanation, prediction, and connections among story events.	Provides contextual support for the earlier gains in higher-level comprehension observed in the dialogic reading condition.
Accessible linguistic and visual support	Teachers identified sign language, key vocabulary, visual representations, and story mapping as useful for accessing and organizing narrative information.	Consistent with the improvement observed across all three instructional conditions.

#### 3.2.1 Active engagement in comprehension

This theme included two subthemes: (a) attention and participation and (b) personal connection and motivation. Teachers consistently described increased student attention and participation during the instructional process. They particularly emphasized students' greater willingness to respond when questions were connected to their own experiences and when instruction involved interactive discussion. One teacher stated,

“*When we asked questions about their own experiences and past experiences, I noticed that they understood better. Question-and-answer activities based on interaction and relating the content to their own experiences were the most effective.”* (T2)

Another teacher observed that participation increased over time:

“*Everyone generally participated. Irmak did not communicate at first, but later she also became involved.”* (T3)

Teachers also reported that personally relevant activities and opportunities to retell or discuss stories increased students' motivation to participate. These observations were broadly consistent with the quantitative findings, which showed earlier and stronger improvement in the Dialogic Reading group (see Table 7).

#### 3.2.2 Development of narrative comprehension

This theme included two subthemes: (a) narrative elements and event organization and (b) main idea and problem–solution comprehension. Teachers reported improvements in students' ability to identify and organize narrative information. They particularly noted greater success in recognizing characters, setting, and the sequence of events when visual supports were provided. One teacher explained,

“*Characters, time, and setting were successful. They analyzed the story better by looking at the visual materials.”* (T4)

However, teachers also identified continued difficulties with higher-level aspects of narrative comprehension, particularly identifying the main idea and understanding relationships among story events. One teacher stated,

“*They can do the sequence of events in the story, but when it comes to the main idea, it is very, very difficult.”* (T3)

Teachers nevertheless perceived improvement over time. As one teacher noted,

“*There was definitely improvement. Students' comprehension abilities developed better.”* (T2)

These observations were particularly consistent with the quantitative pattern for higher-level comprehension, in which the Dialogic Reading condition showed stronger gains in problem, outcome, and main-idea comprehension (see Table 7).

#### 3.2.3 Interaction and instructional scaffolding

This theme included two subthemes: (a) questioning and dialogue and (b) prompting, repetition, and feedback. Teachers emphasized the role of systematic questioning and dialogue in supporting students' comprehension. They reported that questions connected to students' prior knowledge and everyday experiences encouraged students to explain and elaborate on their understanding. One teacher stated,

“*Question-and-answer activities and relating the content to daily life were very important. They understood better.”* (T6)

Teachers also described the use of key words, prompts, and guided support as useful for helping students focus on important information and organize story content. One teacher explained,

“*We tried to relate the text to the children's prior knowledge and provided key words. This was very useful.”* (T3)

Teachers further reported that repeated engagement with the stories and guided retelling helped students organize and summarize narrative information. These observations provide contextual support for the stronger gains in higher-level comprehension observed in the Dialogic Reading condition (see Table 7).

#### 3.2.4 Accessible linguistic and visual support

This theme included two subthemes: (a) sign language and vocabulary support and (b) visual materials and story mapping. Teachers emphasized the importance of accessible linguistic support throughout the instructional process. They particularly noted that students benefited from sign language support when encountering unfamiliar or abstract vocabulary. One teacher explained,

“*They needed sign language support more for words whose meanings they did not know, especially abstract words.”* (T5)

Teachers also highlighted the value of visual materials and story mapping for organizing narrative information. One teacher reported,

“*The story map organized the information and helped them understand the text better. They were able to see the whole passage as a complete structure.”* (T2)

Visual representations were also described as useful for supporting students' understanding of unfamiliar concepts and relationships among story events. These observations help contextualize the improvement observed across all three instructional conditions and highlight the complementary role of accessible language and visual organization in narrative comprehension (see Table 7).

Overall, teachers' accounts indicated improvements in students' engagement and narrative comprehension across the three instructional conditions. Teachers particularly emphasized the value of interactive questioning, personal connections, sign language, visual supports, and story mapping in supporting students' access to and organization of narrative information. The qualitative findings therefore provide contextual rather than confirmatory evidence for the quantitative pattern, particularly the stronger gains observed in higher-level comprehension components in the Dialogic Reading condition.

## 4 Discussion

The present study compared guided reading, dialogic reading, and Sign Language Instruction in relation to narrative comprehension among deaf and hard-of-hearing (DHH) students and examined teachers' perceptions of students' engagement and comprehension processes. Overall, all three instructional conditions were associated with improvement over the intervention period, but the timing and magnitude of change differed across approaches. The dialogic reading condition demonstrated earlier and more pronounced gains, particularly for higher-level narrative comprehension components involving problem identification, outcome, and main-idea construction. These differences were also evident at follow-up, where both the dialogic reading and Sign Language Instruction groups outperformed the guided reading group on the overall comprehension score (three-group omnibus test; ε^2^ = 0.75); the dialogic reading and Sign Language Instruction groups did not differ significantly from each other on this contrast. The particularly large effect observed for main-idea extraction at posttest (ε^2^ = 1.00) further suggests that the potential advantage of dialogic reading was most apparent when comprehension required students to integrate information across multiple narrative events rather than identify explicitly stated details. The qualitative findings complemented this pattern, as teachers described greater participation, more elaborated responses, increased attention to relationships among narrative elements, and the importance of systematic questioning and feedback during instruction.

### 4.1 Dialogic reading and higher-level narrative comprehension

The stronger pattern of improvement associated with dialogic reading may be interpreted in relation to the cognitive and linguistic demands embedded in this instructional approach. Unlike approaches that rely more heavily on teacher explanation or structured guidance, dialogic reading repeatedly positions learners as active participants in meaning construction. In the present study, the use of PEER and CROWD procedures required students to retrieve information, predict events, explain their reasoning, make personal connections, and elaborate on responses. These exchanges provided repeated opportunities to move beyond identification of individual story elements toward establishing relationships among events and constructing an integrated interpretation of the narrative.

This interpretation is consistent with broader evidence on dialogic reading. A recent three-level meta-analysis of 64 studies involving 10,463 children reported significant effects of dialogic reading on reading literacy and reading comprehension, although effect sizes varied according to participant and assessment characteristics (Tang et al., 2026). Importantly, this evidence is derived primarily from hearing children and therefore should not be generalized directly to DHH learners. Nevertheless, it provides broader support for the proposition that the interactive features of dialogic reading can facilitate comprehension development. The present findings extend this literature by demonstrating a similar pattern among DHH students and by showing that the strongest differences were particularly evident in narrative components requiring integration across events.

Evidence specific to DHH learners further supports this interpretation. Fung et al. (2005) reported that an 8-week dialogic reading intervention produced greater gains in receptive vocabulary among DHH children than comparison conditions, suggesting that repeated interaction around text can support language processes relevant to literacy development. Dirks and Wauters (2018) similarly found that training in interactive storybook reading increased the use of interactive reading strategies with young DHH children. Scott and Hansen (2020) also reported promising evidence concerning dialogic reading with a deaf elementary student, emphasizing the potential contribution of prompting and interaction to comprehension of informational text. Taken together, these studies and the present findings suggest that the contribution of dialogic reading may extend beyond increasing exposure to text; repeated opportunities to explain, elaborate, predict, and justify interpretations may provide additional support for constructing meaning from written language.

The pattern observed across narrative components is particularly informative in this respect. Basic structural elements such as setting, time, and character were generally identified at comparable levels across conditions, whereas greater between-group differences emerged for problem, solution, outcome, and main-idea comprehension. This distinction is theoretically meaningful because explicitly stated narrative elements can often be identified from surface-level information, whereas higher-level comprehension requires readers to integrate multiple events into a coherent representation of the narrative (Mandler and Johnson, 1977; Paris and Paris, 2003; Stein and Glenn, 1979). Thus, the stronger performance of the dialogic reading group may reflect the extent to which the instructional process repeatedly required students to make connections among narrative events rather than simply retrieve isolated details.

The qualitative findings provide convergent contextual evidence for this interpretation. Teachers reported that students increasingly established relationships among characters, problems, solutions, and outcomes and became more able to articulate the overall meaning of stories. These observations are consistent with the quantitative finding that the largest differences between conditions were observed in components requiring integration and interpretation. However, because the dialogic condition incorporated multiple interacting instructional features, including open-ended questioning, elaboration, feedback, and discussion, the findings should be interpreted as evidence concerning the effectiveness of the dialogic instructional package rather than the isolated effect of any single questioning technique.

### 4.2 Narrative structure and the role of the story map

The improvement observed across all three conditions also highlights the potential importance of explicitly organizing narrative structure. All participants received instruction using the same story-map framework, which included setting, time, characters, problem, solution, outcome, reaction, and main idea. Consequently, the story map should not be regarded as the experimental factor distinguishing the groups. Instead, it functioned as a common instructional scaffold that may have contributed to the improvement observed across conditions.

From a Story Grammar perspective, narrative comprehension involves not only recognition of individual elements but also understanding the structural and causal relationships among them (Mandler and Johnson, 1977; Stein and Glenn, 1979). The story map operationalized this structure by making key narrative components visually explicit and by providing a consistent organizational framework across sessions. Previous intervention research has similarly suggested that story mapping can facilitate the identification and organization of story-grammar elements and support narrative comprehension (Boulineau et al., 2004). Evidence from broader literacy populations also indicates that explicit visual organization can provide students with a concrete framework for integrating narrative information, particularly when it is accompanied by modeling, questioning, and feedback.

The present qualitative findings further support this interpretation. Teachers described visual representations and the story map as useful for helping students recall important events and connect different elements of the story. Importantly, however, because the story map was implemented in all three conditions, the present study cannot establish its independent causal contribution. The findings instead suggest that a common structural scaffold may have provided a shared foundation for learning, while the nature and intensity of teacher–student interaction may have contributed to the different trajectories observed across instructional conditions. Future studies could isolate this contribution by comparing instruction with and without explicit story mapping or by experimentally manipulating the intensity and timing of story-map use.

### 4.3 Accessible language and sign-language instruction

A second important implication concerns access to meaningful linguistic input. Teachers consistently identified sign language, visual representations, and vocabulary support as important resources for students' access to narrative content. This finding is also consistent with Işikdogan Ugurlu and Kayhan (2025), who highlighted the importance of accessible language and communication support in the education of deaf and hard-of-hearing children from the perspective of special education teacher candidates. Their findings suggest that teachers' awareness of linguistic accessibility and their ability to provide appropriate communication support are important components of educational practice for DHH learners. In the present study, teachers similarly emphasized the importance of sign language, visual supports, and accessible instructional explanations for facilitating students' engagement with narrative content. Together, these findings underscore that effective literacy instruction for DHH learners requires attention not only to the instructional strategy itself but also to the linguistic accessibility of the instructional environment. A recent systematic review of 36 studies found consistent evidence for a positive relationship between lexical skills and text-level reading comprehension among deaf readers and also identified evidence of a cross-modal relationship between sign-language skills and reading comprehension (Olujić Tomazin et al., 2025). These findings reinforce the importance of considering language development and language modality when interpreting reading comprehension among DHH students. This issue is particularly relevant in the Turkish context, where recent studies have identified continuing challenges in teachers' sign-language competence and the consistent integration of sign language into educational practice. For example, Turkish studies have reported that teachers may have limited preparation for working through sign language and may rely on informal learning or additional support, while visual materials and participation-oriented strategies are commonly used in classroom practice (Hasanoglu Yazçayir and Girgin, 2019; Selvi and Yildirim, 2023). More recent evidence from Türkiye similarly indicates that sign language use in schools can be constrained by teacher proficiency and speech-first educational practices (Yazçayir and Holmström, 2026). These findings provide a relevant local context for the present study, in which teachers emphasized sign language, visual supports, and accessible explanations as important resources for narrative comprehension.

The Turkish context also highlights the importance of considering language development from the early intervention period onward. Recent research in Türkiye has identified variability in family-centered early intervention practices, including limited parental involvement, inconsistent intervention practices, and difficulties in accessing systematic support for language development (Dogan et al., 2026). Similarly, early-auditory intervention has been associated with better receptive and expressive language outcomes among children without additional disabilities when intervention begins earlier (Çelik et al., 2021). Together, these findings suggest that later literacy instruction should be considered within a broader developmental pathway in which early access to language, family support, and sustained educational opportunities may shape subsequent engagement with written language.

The present findings are also consistent with recent reviews of literacy research involving signed languages. Dostal et al. (2024) identified substantial variation in the ways signed language is incorporated into literacy education across contexts, while emphasizing the importance of accessible language and instructional approaches that recognize the linguistic resources of signing deaf learners. More recently, Dostal et al. (2025) reviewed 15 empirical studies of literacy interventions using signed languages and reported promising evidence concerning literacy learning while also highlighting considerable methodological heterogeneity in the existing evidence base. Thus, the improvement observed in the direct instruction through sign language condition should not be understood simply as the result of translating written information into sign language. Rather, accessible linguistic input may provide an essential foundation through which students can organize, interpret, and discuss written information.

At the same time, the stronger performance of the dialogic reading group indicates that linguistic accessibility alone may not fully account for higher-level comprehension development. In the sign-language condition, teachers explicitly modeled narrative components and provided explanations, prompts, and feedback, thereby providing substantial access to the content. Nevertheless, the earliest and strongest gains in problem identification and main-idea construction were observed in the dialogic reading condition. This pattern suggests that accessible linguistic input may constitute an enabling condition for comprehension, whereas opportunities to actively use that input through explanation, inference, prediction, elaboration, and causal reasoning may provide additional support for higher-level narrative processing. The distinction is important because it positions language accessibility and cognitive engagement as complementary rather than competing instructional priorities.

### 4.4 Guided reading and gradual development

The findings for guided reading provide an important counterpoint to the stronger pattern observed for dialogic reading. Guided reading was associated with meaningful improvement over time, although gains in higher-level narrative components appeared later than those observed in the dialogic condition. This pattern should not be interpreted as evidence that guided reading was ineffective. Rather, it suggests that different instructional approaches may support similar broad comprehension goals through different pathways and at different rates.

The guided reading intervention incorporated prediction, vocabulary support, comprehension questions, teacher prompts, and feedback, providing students with systematic scaffolding while maintaining a structured instructional sequence. Such support may be particularly valuable when learners require explicit guidance in organizing narrative information and gradually assuming greater responsibility for comprehension processes. The delayed emergence of gains in higher-level components may therefore reflect a more gradual development of these processes rather than an absence of instructional benefit.

This interpretation is supported by the overall trajectory of the quantitative findings. All three groups demonstrated improvement over time, while the principal difference among conditions concerned the timing and magnitude of change. The qualitative findings were similarly consistent with improvement across groups, with teachers reporting increased ability to identify and organize narrative information. Thus, the findings do not suggest that one instructional approach universally replaces the others. Instead, they indicate that dialogic reading may be particularly advantageous when the instructional goal is to accelerate active meaning construction and higher-level integration, whereas guided reading may provide a structured pathway for progressively developing comprehension skills.

### 4.5 Integration of quantitative and qualitative findings

The mixed-methods findings are important because the two strands of evidence addressed complementary aspects of the instructional process. The quantitative findings established that comprehension improved across all three conditions and that dialogic reading was associated with stronger and earlier gains in several higher-level narrative components. The qualitative findings provided contextual information about how teachers perceived students' participation, responses, and use of narrative information during instruction. As summarized in Table 7, teachers associated dialogic reading with greater participation and attention, narrative comprehension with improved organization of story information, instructional scaffolding with deeper processing, and accessible linguistic and visual supports with improved access to textual information.

The convergence between the two strands is particularly relevant to interpretation of the mechanisms underlying the quantitative pattern. Earlier gains in problem identification and main-idea comprehension coincided with teachers' observations of increased questioning, elaboration, discussion, and attention to relationships among narrative elements. This convergence does not establish a causal mechanism, because the qualitative data were based on teachers' perceptions rather than independent observation or experimental manipulation of instructional processes. Nevertheless, the qualitative findings provide a plausible explanatory account that is consistent with the quantitative trajectories. In this sense, the mixed-methods design strengthens interpretation by showing not only that performance differed across conditions but also how instructional processes may have contributed to those differences.

The qualitative findings should therefore be viewed as explanatory rather than confirmatory evidence of intervention effectiveness. Only six teachers participated in the interviews, and their perceptions may have been influenced by their experiences with particular instructional conditions. Future research would benefit from combining teacher reports with systematic observations, video-based coding of teacher–student interaction, and direct measures of student engagement and verbal or signed elaboration.

### 4.6 Theoretical implications

The findings have theoretical implications for understanding narrative comprehension among DHH learners. First, they provide support for interpreting narrative comprehension through the complementary perspectives of Story Grammar and socially mediated learning. Story Grammar emphasizes the organization of narratives around structural and causal relationships (Mandler and Johnson, 1977; Stein and Glenn, 1979), whereas a socially mediated perspective emphasizes the role of interaction, questioning, feedback, and scaffolding in supporting learners' construction of meaning. The present findings suggest that these perspectives can be integrated: visual and linguistic supports may facilitate access to narrative information, while dialogic interaction may provide opportunities for students to reorganize, elaborate, and integrate that information.

Second, the findings contribute to a broader conceptualization of literacy instruction for DHH students in which language accessibility and active cognitive participation are considered interdependent. Contemporary evidence indicates that both spoken-language and sign-language competencies can be associated with text-level reading comprehension among deaf readers (Olujić Tomazin et al., 2025). Similarly, research on signed-language-supported literacy interventions emphasizes the importance of providing accessible linguistic pathways to literacy learning (Dostal et al., 2024, 2025). The present findings extend this perspective by suggesting that access to language may be most productive when learners are also given repeated opportunities to use that language for reasoning, explanation, prediction, and integration of information.

Theoretically, therefore, the findings support a distinction between access to narrative information and active construction of narrative meaning. The three instructional conditions shared features that promoted access, including visual supports, structured narrative organization, and language support. Dialogic reading added more frequent opportunities for students to actively process and articulate relationships among narrative elements. This distinction offers a potential framework for understanding why all three conditions supported improvement while dialogic reading showed a stronger pattern for higher-level narrative comprehension.

### 4.7 Implications for practice

The findings have several implications for literacy instruction with DHH students. First, instruction should provide consistent access to meaningful linguistic and visual information through sign language when appropriate, visual representations, explicit vocabulary support, and clearly structured narrative materials. Second, explicit attention to narrative structure may help students organize information across events and develop a coherent representation of the story. Third, teachers may benefit from incorporating dialogic questioning that moves beyond factual recall toward prediction, explanation, justification, causal reasoning, and connections between text and prior experience.

The findings also suggest that dialogic interaction should not be implemented as a replacement for accessible language support. Rather, the most productive instructional framework may combine accessible linguistic input with cognitively demanding interaction. In practice, this may involve presenting narrative information through accessible modalities, explicitly organizing story structure, and then using open-ended questions and feedback to encourage students to explain how and why events are related. Such an integrated approach may be particularly useful when instructional goals extend beyond recall to inferential and higher-level narrative comprehension.

In the Turkish context, these findings also support the need for classroom practices and educational resources that combine linguistic accessibility with active comprehension-oriented interaction. This is consistent with recent developments in Türkiye, including the expansion of Turkish Sign Language within the curriculum, while also highlighting the importance of teacher competence, accessible instructional materials, and sustained professional development. Thus, the findings have implications not only for classroom-level literacy instruction but also for the implementation of inclusive and linguistically accessible educational policies for DHH students.

## 5 Limitations and directions for future research

Several limitations should be considered when interpreting the findings. First, the sample consisted of 30 DHH students, with 10 students in each instructional condition. Although participants were randomly assigned to the three conditions, the small number of participants limits statistical power and the generalizability of the findings. Replication with larger and more diverse samples is therefore necessary.

Second, the intervention lasted 8 weeks, and the follow-up assessment was conducted only 1 week after the posttest. Accordingly, the findings provide evidence of short-term maintenance but cannot establish the long-term durability of the observed gains. Longer follow-up periods are needed to determine whether the advantages associated with dialogic reading persist over subsequent months.

Third, the outcome measure focused on narrative comprehension through story-map components. Although this provided a detailed examination of specific narrative processes, it does not represent the full construct of reading comprehension. In particular, the measure did not directly assess comprehension of expository texts, broader inferential processing, vocabulary development as an independent outcome, or transfer to independent reading. Future research should therefore incorporate multiple measures covering narrative and expository comprehension, vocabulary, language proficiency, inference, and transfer.

Fourth, the qualitative component involved six teachers and relied on teacher perceptions rather than systematic independent observation of student behavior. Although these data provided useful contextual information, future mixed-methods studies should incorporate direct observations, video-based interaction coding, and multiple sources of qualitative evidence.

Fifth, because instruction was delivered in instructional dyads and data were primarily analyzed through separate non-parametric tests rather than a comprehensive multi-level interaction model, formal simultaneous modeling of the dyadic nesting structure and Group × Time interaction terms was not fully executed. Although weekly Kruskal-Wallis tests and within-group Friedman analyses were utilized to track temporal progress and differential trajectories, future research with larger samples should employ Generalized Linear Mixed Models (GLMM) or Generalized Estimating Equations (GEE) to explicitly account for clustering and multi-point interaction matrices.

Finally, because the story map and several visual supports were shared across all three conditions, the independent contributions of these components cannot be disentangled from the effects of the instructional approaches. In addition, the dialogic condition incorporated several related instructional features, including open-ended questioning, elaboration, feedback, and discussion. Consequently, the present study cannot determine whether the observed advantage was attributable primarily to dialogic questioning itself or to the combined instructional package. Future research should experimentally manipulate the amount and type of dialogic interaction, the use of story mapping, and the intensity of linguistic and visual support. Larger randomized studies with longer follow-up periods and process-level measures of teacher–student interaction would be particularly valuable for identifying the mechanisms through which different instructional approaches influence narrative comprehension among DHH learners.

Overall, the findings suggest that guided reading, dialogic reading, and direct instruction through sign language can all support the development of narrative comprehension, but they may do so through different pathways. Accessible linguistic and visual supports appear to provide an important foundation for understanding narrative information, whereas dialogic interaction may provide additional opportunities for learners to explain, integrate, and elaborate relationships among narrative events. The stronger pattern of higher-level comprehension observed in the dialogic reading condition therefore highlights the potential value of combining linguistic accessibility with cognitively demanding interaction in literacy instruction for DHH students.

## Data Availability

The original contributions presented in the study are included in the article/Supplementary material, further inquiries can be directed to the corresponding author.

## Appendix A

**Table A1:** Sample PEER and CROWD questions used during dialogic reading sessions.

Strategy	Purpose	Example Question from the Story “Respect for the Elderly”
Completion (C)	Encourage students to complete a sentence or idea from the story	“Fatih stood up and gave his seat to the ______.”
Recall (R)	Encourage students to remember story events	“What did Fatih see after he got on the bus?”
Open-ended (O)	Encourage students to describe events and elaborate on ideas	“What is happening in this picture?”
Wh- Questions (W)	Promote comprehension through who, what, where, when, why, and how questions	“Why did Fatih give his seat to the elderly woman?”
Distancing (D)	Relate story events to students' personal experiences	“Have you ever helped an elderly person? What did you do?”
Prompt (P)	Encourage students to respond to a question or comment	“What do you think Fatih should do when he sees the elderly woman standing?”
Evaluate (E)	Provide feedback on the student's response	“Yes, that's correct. Fatih noticed that the elderly woman was having difficulty standing.”
Expand (E)	Expand the student's response by adding information	Student: “He gave his seat.” Teacher: “Yes, he gave his seat because he wanted to help the elderly woman.”
Repeat (R)	Encourage the student to repeat the expanded response	“Can you say that again? Why did Fatih give his seat to the elderly woman?”
