# Supplementary material for: Comparing guided, dialogic, and sign language instruction in deaf and hard-of-hearing students' narrative comprehension
Source: Front Psychol. 2026 Oct 20;17:1982563. doi: 10.3389/fpsyg.2026.1982563 (PMC13727612; doi:10.3389/fpsyg.2026.1982563)
Supplement: Supplementary file 1 [file Supplementary_file_1.docx]

# SUPPLEMENTARY MATERIALS: Story Components Comprehensive Analysis Tables

The following tables present the Kruskal-Wallis H test findings and Friedman Test time-dependent progress analyses of the story components obtained during the pre-test, eight-week implementation (Weeks 1-8), post-test, and follow-up measurements of the reading comprehension intervention process.

Effect Size (ε²) Interpretation Guide: ε² < .01 = Very Small; .01-.06 = Small; .06-.14 = Medium; > .14 = Large.

Significance Level: * p < .05, ** p < .01, *** p < .001

Abbreviations: GR = Guided Reading, DR = Dialogic Reading, SLI = Sign Language Instruction

## Supplementary Table 1: *Pre-test (Pre-evaluation) Measurement*

| Component | GR (Mrank) | DR (Mrank) | SLI (Mrank) | H | p | ε² | Effect Level | Significant Difference |
| --- | --- | --- | --- | --- | --- | --- | --- | --- |
| Pre-test Setting | 15.50 | 17.00 | 14.00 | 0.81 | .668 | -.04 | Very Small | None |
| Pre-test Time | 15.00 | 15.00 | 16.50 | 0.36 | .835 | -.06 | Very Small | None |
| Pre-test Character | 15.00 | 16.50 | 15.00 | 0.33 | .848 | -.06 | Very Small | None |
| Pre-test Problem | 15.50 | 15.50 | 15.50 | 0.00 | 1.000 | -.07 | Very Small | None |
| Pre-test Outcome | 15.50 | 15.50 | 15.50 | 0.00 | 1.000 | -.07 | Very Small | None |
| Pre-test Conclusion | 15.50 | 15.50 | 15.50 | 0.00 | 1.000 | -.07 | Very Small | None |
| Pre-test Reaction | 14.00 | 15.50 | 17.00 | 0.92 | .631 | -.04 | Very Small | None |
| Pre-test Main Idea | 15.50 | 15.50 | 15.50 | 0.00 | 1.000 | -.07 | Very Small | None |

## Supplementary Table 2: *Week 1 - Statistical Comparison of All Story Components*

| Component | GR (Mrank) | DR (Mrank) | SLI (Mrank) | H | p | ε² | Effect Level | Significant Difference |
| --- | --- | --- | --- | --- | --- | --- | --- | --- |
| Week 1 Setting | 16.50 | 15.00 | 15.00 | 2.00 | .368 | -.04 | Very Small | None |
| Week 1 Time | 13.50 | 15.00 | 18.00 | 2.03 | .362 | -.04 | Very Small | None |
| Week 1 Character | 16.50 | 15.00 | 15.00 | 2.00 | .368 | -.04 | Very Small | None |
| Week 1 Problem | 13.50 | 16.50 | 16.50 | 2.23 | .328 | -.03 | Very Small | None |
| Week 1 Resolution | 13.50 | 16.50 | 16.50 | 2.23 | .328 | -.03 | Very Small | None |
| Week 1 Conclusion | 13.50 | 16.50 | 16.50 | 2.23 | .328 | -.03 | Very Small | None |
| Week 1 Reaction | 12.50 | 17.00 | 17.00 | 2.42 | .299 | -.02 | Very Small | None |
| Week 1 Main Idea | 12.50 | 17.00 | 17.00 | 3.63 | .163 | .02 | Very Small | None |

## Supplementary Table 3: *Week 2 - Statistical Comparison of All Story Components*

| Component | GR (Mrank) | DR (Mrank) | SLI (Mrank) | H | p | ε² | Effect Level | Significant Difference |
| --- | --- | --- | --- | --- | --- | --- | --- | --- |
| Week 2 Setting | 13.50 | 18.00 | 15.00 | 2.52 | .283 | .02 | Very Small | None |
| Week 2 Time | 15.50 | 15.50 | 15.50 | 0.00 | 1.000 | -.07 | Very Small | None |
| Week 2 Character | 15.00 | 16.50 | 15.00 | 0.56 | .757 | -.05 | Very Small | None |
| Week 2 Problem | 10.00 | 17.50 | 19.00 | 8.03 | .018* | .22 | Medium | SLI > GR* |
| Week 2 Resolution | 12.00 | 16.50 | 18.00 | 3.77 | .152 | .07 | Medium | None |
| Week 2 Conclusion | 12.00 | 16.50 | 18.00 | 4.68 | .096 | .10 | Medium | None |
| Week 2 Reaction | 8.30 | 19.10 | 19.10 | 12.00 | .002** | .41 | Large | DR > GR**, SLI > GR** |
| Week 2 Main Idea | 7.00 | 19.45 | 20.05 | 18.25 | .000*** | .64 | Large | DR > GR***, SLI > GR*** |

## Supplementary Table 4: *Week 3 - Statistical Comparison of All Story Components*

| Component | GR (Mrank) | DR (Mrank) | SLI (Mrank) | H | p | ε² | Effect Level | Significant Difference |
| --- | --- | --- | --- | --- | --- | --- | --- | --- |
| Week 3 Setting | 18.00 | 19.50 | 9.00 | 14.17 | .001** | .49 | Large | GR > SLI** |
| Week 3 Time | 17.00 | 17.00 | 12.50 | 2.42 | .299 | .02 | Very Small | None |
| Week 3 Character | 16.50 | 16.50 | 13.50 | 1.32 | .517 | -.03 | Very Small | None |
| Week 3 Problem | 6.00 | 20.05 | 20.45 | 21.88 | .000*** | .74 | Large | DR > GR***, SLI > GR*** |
| Week 3 Resolution | 9.00 | 18.05 | 19.45 | 10.79 | .005** | .36 | Large | DR > GR**, SLI > GR** |
| Week 3 Conclusion | 8.50 | 17.50 | 20.50 | 13.46 | .001** | .46 | Large | DR > GR***, SLI > GR*** |
| Week 3 Reaction | 12.00 | 18.00 | 16.50 | 4.68 | .096 | .10 | Medium | None |
| Week 3 Main Idea | 6.50 | 20.00 | 20.00 | 19.97 | .000*** | .70 | Large | DR > GR***, SLI > GR*** |

## Supplementary Table 5: *Week 4 - Statistical Comparison of All Story Components*

| Component | GR (Mrank) | DR (Mrank) | SLI (Mrank) | H | p | ε² | Effect Level | Significant Difference |
| --- | --- | --- | --- | --- | --- | --- | --- | --- |
| Week 4 Setting | 8.00 | 23.00 | 15.50 | 19.33 | .000*** | .68 | Large | DR > GR*** |
| Week 4 Time | 11.00 | 21.50 | 14.00 | 11.97 | .003** | .41 | Large | DR > GR*** |
| Week 4 Character | 16.00 | 17.50 | 13.00 | 3.90 | .142 | .07 | Medium | None |
| Week 4 Problem | 10.50 | 21.00 | 15.00 | 10.73 | .005** | .36 | Large | DR > GR** |
| Week 4 Resolution | 12.50 | 17.15 | 16.85 | 2.95 | .229 | .04 | Small | None |
| Week 4 Conclusion | 13.50 | 18.00 | 15.00 | 3.90 | .142 | .07 | Medium | None |
| Week 4 Reaction | 16.00 | 17.50 | 13.00 | 3.90 | .142 | .07 | Medium | None |
| Week 4 Main Idea | 6.50 | 21.35 | 18.65 | 18.46 | .000*** | .65 | Large | DR > GR***, SLI > GR*** |

## Supplementary Table 6: *Week 5 - Statistical Comparison of All Story Components*

| Component | GR (Mrank) | DR (Mrank) | SLI (Mrank) | H | p | ε² | Effect Level | Significant Difference |
| --- | --- | --- | --- | --- | --- | --- | --- | --- |
| Week 5 Setting | 17.00 | 18.50 | 11.00 | 8.46 | .015* | .24 | Large | DR > SLI* |
| Week 5 Time | 14.50 | 19.00 | 13.00 | 3.41 | .182 | .05 | Medium | None |
| Week 5 Character | 15.50 | 17.00 | 14.00 | 2.15 | .342 | .01 | Very Small | None |
| Week 5 Problem | 10.50 | 21.00 | 15.00 | 10.73 | .005** | .36 | Large | DR > GR** |
| Week 5 Resolution | 10.90 | 19.25 | 16.35 | 7.26 | .026* | .19 | Large | DR > GR* |
| Week 5 Conclusion | 10.00 | 22.60 | 13.90 | 15.13 | .001** | .49 | Large | DR > GR***, DR > SLI** |
| Week 5 Reaction | 10.00 | 19.00 | 17.50 | 8.60 | .014* | .24 | Large | DR > GR* |
| Week 5 Main Idea | 8.00 | 23.00 | 15.50 | 17.40 | .000*** | .57 | Large | DR > GR*** |

## Supplementary Table 7: *Week 6 - Statistical Comparison of All Story Components*

| Component | GR (Mrank) | DR (Mrank) | SLI (Mrank) | H | p | ε² | Effect Level | Significant Difference |
| --- | --- | --- | --- | --- | --- | --- | --- | --- |
| Week 6 Setting | 10.00 | 20.50 | 16.00 | 10.27 | .006** | .34 | Large | DR > GR** |
| Week 6 Time | 14.50 | 20.50 | 11.50 | 8.12 | .017* | .23 | Large | DR > SLI* |
| Week 6 Character | 14.50 | 16.00 | 16.00 | 0.56 | .757 | -.05 | Very Small | None |
| Week 6 Problem | 17.00 | 20.00 | 9.50 | 10.05 | .007** | .34 | Large | DR > SLI** |
| Week 6 Resolution | 9.55 | 23.55 | 13.40 | 18.35 | .000*** | .64 | Large | DR > GR***, DR > SLI*** |
| Week 6 Conclusion | 9.50 | 21.50 | 15.50 | 12.89 | .002** | .44 | Large | DR > GR*** |
| Week 6 Reaction | 16.50 | 16.50 | 13.50 | 4.14 | .126 | .08 | Medium | None |
| Week 6 Main Idea | 7.25 | 21.20 | 16.72 | 17.09 | .000*** | .62 | Large | DR > GR***, SLI > GR** |

## Supplementary Table 8: Week 7 - Statistical Comparison of All Story Components

| Component | GR (Mrank) | DR (Mrank) | SLI (Mrank) | H | p | ε² | Effect Level | Significant Difference |
| --- | --- | --- | --- | --- | --- | --- | --- | --- |
| Week 7 Setting | 16.50 | 16.50 | 13.50 | 4.14 | .126 | .08 | Medium | None |
| Week 7 Time | 14.50 | 19.00 | 13.00 | 4.68 | .096 | .10 | Medium | None |
| Week 7 Character | 16.00 | 16.00 | 14.50 | 2.00 | .368 | -.04 | Very Small | None |
| Week 7 Problem | 15.50 | 18.50 | 12.50 | 4.83 | .089 | .11 | Medium | None |
| Week 7 Resolution | 9.00 | 22.50 | 15.00 | 16.01 | .000*** | .52 | Large | DR > GR***, DR > SLI |
| Week 7 Conclusion | 16.50 | 19.50 | 10.50 | 7.77 | .021* | .21 | Large | DR > SLI* |
| Week 7 Reaction | 14.00 | 17.00 | 15.50 | 2.15 | .342 | .01 | Very Small | None |
| Week 7 Main Idea | 9.10 | 23.05 | 14.35 | 16.97 | .000*** | .55 | Large | DR > GR***, DR > SLI* |

## Supplementary Table 9: *Week 8 - Statistical Comparison of All Story Components*

| Component | GR (Mrank) | DR (Mrank) | SLI (Mrank) | H | p | ε² | Effect Level | Significant Difference |
| --- | --- | --- | --- | --- | --- | --- | --- | --- |
| Week 8 Setting | 13.50 | 16.50 | 16.50 | 4.14 | .126 | .08 | Medium | None |
| Week 8 Time | 14.00 | 17.00 | 15.50 | 2.15 | .342 | .01 | Very Small | None |
| Week 8 Character | 15.50 | 15.50 | 15.50 | 0.00 | 1.000 | -.07 | Very Small | None |
| Week 8 Problem | 16.00 | 16.00 | 14.50 | 2.00 | .368 | -.04 | Very Small | None |
| Week 8 Resolution | 8.50 | 20.50 | 17.50 | 13.46 | .001** | .46 | Large | DR > GR**, SLI > GR** |
| Week 8 Conclusion | 14.50 | 16.00 | 16.00 | 2.00 | .368 | -.04 | Very Small | None |
| Week 8 Reaction | 15.50 | 15.50 | 15.50 | 0.00 | 1.000 | -.07 | Very Small | None |
| Week 8 Main Idea | 10.00 | 22.00 | 14.50 | 12.86 | .002** | .44 | Large | DR > GR**, DR > SLI |

## Supplementary Table 10: *Post-test Measurement (10 Weeks After Intervention)*

| Component | GR (Mrank) | DR (Mrank) | SLI (Mrank) | H | p | ε² | Effect Level | Significant Difference |
| --- | --- | --- | --- | --- | --- | --- | --- | --- |
| Post-test Setting | 15.00 | 16.50 | 15.00 | 1.04 | .596 | -.07 | Very Small | None |
| Post-test Time | 14.50 | 16.00 | 16.00 | 2.00 | .368 | -.04 | Very Small | None |
| Post-test Character | 15.50 | 15.50 | 15.50 | 0.00 | 1.000 | -.07 | Very Small | None |
| Post-test Problem | 7.50 | 21.00 | 18.00 | 18.59 | .000*** | .65 | Large | DR > GR***, SLI > GR*** |
| Post-test Resolution | 15.00 | 16.50 | 15.00 | 1.04 | .596 | -.07 | Very Small | None |
| Post-test Conclusion | 15.50 | 15.50 | 15.50 | 0.00 | 1.000 | -.07 | Very Small | None |
| Post-test Reaction | 13.50 | 16.50 | 16.50 | 4.14 | .126 | .08 | Medium | None |
| Post-test Main Idea | 5.50 | 20.50 | 20.50 | 29.00 | .000*** | 1.00 | Very Large | DR > GR***, SLI > GR*** |

## Supplementary Table 11: *Follow-up Measurement (1 Week After Post-test)*

| Component | GR (Mrank) | DR (Mrank) | SLI (Mrank) | H | p | ε² | Effect Level | Significant Difference |
| --- | --- | --- | --- | --- | --- | --- | --- | --- |
| Follow-up Setting | 16.00 | 16.00 | 14.50 | 2.00 | .368 | -.04 | Very Small | None |
| Follow-up Time | 15.50 | 15.50 | 15.50 | 0.00 | 1.000 | -.07 | Very Small | None |
| Follow-up Character | 15.50 | 15.50 | 15.50 | 0.00 | 1.000 | -.07 | Very Small | None |
| Follow-up Problem | 8.00 | 21.50 | 17.00 | 16.24 | .000*** | .56 | Large | DR > GR***, SLI > GR* |
| Follow-up Resolution | 17.00 | 17.00 | 12.50 | 6.44 | .040* | .16 | Large | DR > SLI* |
| Follow-up Conclusion | 17.00 | 18.50 | 11.00 | 8.46 | .015* | .24 | Large | DR > SLI*, GR > SLI |
| Follow-up Reaction | 6.50 | 20.00 | 20.00 | 24.86 | .000*** | .88 | Very Large | DR > GR***, SLI > GR*** |
| Follow-up Main Idea | 7.00 | 22.00 | 17.50 | 20.73 | .000*** | .73 | Large | DR > GR***, SLI > GR** |

## Supplementary Table 12: *General Effect Sizes of Teaching Methods Across Intervention Periods (ε²)*

| Period | Dialogic Reading (DR) | Sign Language Inst. (SLI) | Guided Reading (GR) |
| --- | --- | --- | --- |
| Week 1-3 (Adaptation Phase) | ε² = .35 | ε² = .17 | ε² = 0.08 |
| Week 4-8 (Intervention Phase) | ε² = .62 (Large) | ε² = .41 (Medium) | ε² = .19 (Small) |
| Post test (Retention) | ε² = .65 (Large) | ε² = .65 (Large) | ε² = .58 (Large) |
| Follow up (Long Term) | ε² = .73 (Large) | ε² = .56 (Large) | ε² = .52 (Large) |

## Supplementary Table 13: *Setting Component Time-Dependent Progress Analysis (Friedman Test)*

| Instruction Type | n | df | χ² | p | ε² | Significant Differences (Bonferroni Corrected p < .05) |
| --- | --- | --- | --- | --- | --- | --- |
| Guided Reading | 10 | 10 | 83.41 | .000*** | .83 | Pretest-Based: W3 > Pre-test, W5 > Pre-test, W7 > Pre-test, W8 > Pre-test, Post-test > Pre-test, Follow-up > Pre-test Development-Based: W7 > W1, W7 > W4, W7 > W6, Follow-up > W1, Follow-up > W4, Follow-up > W6 |
| Dialogic Reading | 10 | 10 | 82.95 | .000*** | .83 | Pretest-Based: W3 > Pre-test, W4 > Pre-test, W5 > Pre-test, W7 > Pre-test, W8 > Pre-test, Post-test > Pre-test, Follow-up > Pre-test Development-Based: W3 > W1, W4 > W1, W5 > W1, W7 > W1, W8 > W1, Post-test > W1, Follow-up > W1 |
| Sign Language Inst. | 10 | 10 | 54.55 | .000*** | 0.55 | Pretest-Based: W7 > Pre-test, W8 > Pre-test, Post-test > Pre-test, Follow-up > Pre-test Development-Based: W8 > W1 |

## Supplementary Table 14: *Time Component Time-Dependent Progress Analysis (Friedman Test)*

| Instruction Type | n | df | χ² | p | Kendall's W | Significant Differences (Bonferroni Corrected p < .05) |
| --- | --- | --- | --- | --- | --- | --- |
| Guided Reading | 10 | 10 | 70.63 | .000*** | .11 | Pretest-Based: W6 > Pre-test, W7 > Pre-test, W3 > Pre-test, W8 > Pre-test, Post-test > Pre-test, Follow-up > Pre-test Development-Based: W6 > W1, W3 > W1, W7 > W1, W8 > W1, Post-test > W1, Follow-up > W1 |
| Dialogic Reading | 10 | 10 | 81.64 | .000*** | .82 | Pretest-Based: W5 > Pre-test, W6 > Pre-test, W7 > Pre-test, W8 > Pre-test, Post-test > Pre-test, Follow-up > Pre-test Development-Based: W5 > W1, W6 > W1, W7 > W1, W8 > W1, Post-test > W1, Follow-up > W1 |
| Sign Language Inst. | 10 | 10 | 64.64 | .000*** | .65 | Pretest-Based: W7 > Pre-test, W8 > Pre-test, Post-test > Pre-test, Follow-up > Pre-test Development-Based: W8 > W1, Post-test > W1, Follow-up > W1 |

## Supplementary Table 15: *Character Component Time-Dependent Progress Analysis (Friedman Test)*

| Instruction Type | n | df | χ² | p | ε² | Significant Differences (Bonferroni Corrected p < .05) |
| --- | --- | --- | --- | --- | --- | --- |
| Guided Reading | 10 | 10 | 74.13 | .000*** | .74 | Pretest-Based: W5 > Pre-test, W4 > Pre-test, W7 > Pre-test, W8 > Pre-test, Post-test > Pre-test, Follow-up > Pre-test |
| Dialogic Reading | 10 | 10 | 83.03 | .000*** | .83 | Pretest-Based: W6 > Pre-test, W4 > Pre-test, W8 > Pre-test, W7 > Pre-test, W5 > Pre-test, Post-test > Pre-test, Follow-up > Pre-test Development-Based: W4 > W1, W5 > W1, W7 > W1, W8 > W1, Post-test > W1, Follow-up > W1 |
| Sign Language Inst. | 10 | 10 | 70.16 | .000*** | .70 | Pretest-Based: W7 > Pre-test, W6 > Pre-test, W8 > Pre-test, Post-test > Pre-test, Follow-up > Pre-test Development-Based: W8 > W1, Post-test > W1, Follow-up > W1 |

## Supplementary Table 16: *Problem Component Time-Dependent Progress Analysis (Friedman Test)*

| Instruction Type | n | df | χ² | p | ε² | Significant Differences (Bonferroni Corrected p < .05) |
| --- | --- | --- | --- | --- | --- | --- |
| Guided Reading | 10 | 10 | 90.92 | .000*** | .91 | Pretest/First Phase-Based: W6 > Pre-test, W7 > Pre-test, W8 > Pre-test W6 > W1, W7 > W1, W8 > W1 W6 > W2, W7 > W2, W8 > W2 W6 > W3, W7 > W3, W8 > W3 |
| Dialogic Reading | 10 | 10 | 80.92 | .000*** | .81 | Pretest-Based: W4 > Pre-test, W5 > Pre-test, W6 > Pre-test, Follow-up > Pre-test, W8 > Pre-test, W7 > Pre-test, Post-test > Pre-test Development-Based: W4 > W1, W5 > W1, W6 > W1, Follow-up > W1, W7 > W1, W8 > W1, Post-test > W1 W8 > W2, W7 > W2, Post-test > W2 |
| Sign Language Inst. | 10 | 10 | 68.03 | .000*** | .68 | Pretest-Based: Follow-up > Pre-test, W7 > Pre-test, Post-test > Pre-test, W8 > Pre-test Development-Based: Follow-up > W1, W7 > W1, Post-test > W1, W8 > W1 W8 > W2 |

## Supplementary Table 17: *Resolution Component Time-Dependent Progress Analysis (Friedman Test)*

| Instruction Type | n | df | χ² | p | ε² | Significant Differences (Bonferroni Corrected p < .05) |
| --- | --- | --- | --- | --- | --- | --- |
| Guided Reading | 10 | 10 | 94.21 | .000*** | .94 | First Phase/Pretest-Based: Post-test > Pre-test, Follow-up > Pre-test Post-test > W1, Follow-up > W1 Post-test > W2, Follow-up > W2 Post-test > W3, Follow-up > W3 |
| Dialogic Reading | 10 | 10 | 81.07 | .000*** | .81 | Pretest-Based: W5 > Pre-test, W8 > Pre-test, W7 > Pre-test, W6 > Pre-test, Post-test > Pre-test, Follow-up > Pre-test Development-Based: W8 > W1, W6 > W1, W7 > W1, Post-test > W1, Follow-up > W1 W7 > W2, W6 > W2, Post-test > W2, Follow-up > W2 Post-test > W3, Follow-up > W3 |
| Sign Language Inst. | 10 | 10 | 65.11 | .000*** | .65 | Pretest-Based: W5 > Pre-test, W4 > Pre-test, W7 > Pre-test, W8 > Pre-test, Follow-up > Pre-test, Post-test > Pre-test Development-Based: W8 > W1, Follow-up > W1, Post-test > W1 Post-test > W2, Post-test > W3 |

## Supplementary Table 18: *Conclusion Component Time-Dependent Progress Analysis (Friedman Test)*

| Instruction Type | n | df | χ² | p | Kendall's W | Significant Differences (Bonferroni Corrected p < .05) |
| --- | --- | --- | --- | --- | --- | --- |
| Guided Reading | 10 | 10 | 94.48 | .000*** | .94 | Pretest/First Phase-Based: W7 > Pre-test, Post-test > Pre-test, Follow-up > Pre-test, W8 > Pre-test W7 > W1, Post-test > W1, Follow-up > W1, W8 > W1 W7 > W2, Post-test > W2, Follow-up > W2, W8 > W2 W7 > W3, Post-test > W3, Follow-up > W3, W8 > W3 |
| Dialogic Reading | 10 | 10 | 89.35 | .000*** | .89 | Pretest-Based: W5 > Pre-test, W6 > Pre-test, Post-test > Pre-test, W7 > Pre-test, Follow-up > Pre-test, W8 > Pre-test Development-Based: W5 > W1, W6 > W1, Post-test > W1, W7 > W1, Follow-up > W1, W8 > W1 W5 > W2, W6 > W2, Post-test > W2, W7 > W2, Follow-up > W2, W8 > W2 W7 > W3, Follow-up > W3, W8 > W3 |
| Sign Language Inst. | 10 | 10 | 76.16 | .000*** | .76 | Pretest-Based: W7 > Pre-test, W6 > Pre-test, Follow-up > Pre-test, Post-test > Pre-test, W8 > Pre-test Development-Based: W6 > W1, Follow-up > W1, Post-test > W1, W8 > W1 W8 > W3 |

## Supplementary Table 19: *Reaction Component Time-Dependent Progress Analysis (Friedman Test)*

| Instruction Type | n | df | χ² | p | ε² | Significant Differences (Bonferroni Corrected p < .05) |
| --- | --- | --- | --- | --- | --- | --- |
| Guided Reading | 10 | 10 | 86.31 | .000*** | .86 | Pretest-Based: W7 > Pre-test, Post-test > Pre-test, W4 > Pre-test, W6 > Pre-test, W8 > Pre-test Development-Based: W7 > W1, Post-test > W1, W4 > W1, W6 > W1, W8 > W1 W7 > W2, Post-test > W2, W4 > W2, W6 > W2, W8 > W2 |
| Dialogic Reading | 10 | 10 | 82.48 | .000*** | .82 | Pretest-Based: Follow-up > Pre-test, W8 > Pre-test, W7 > Pre-test, W6 > Pre-test, W4 > Pre-test, Post-test > Pre-test Development-Based: W4 > W1, W6 > W1, W7 > W1, W8 > W1, Post-test > W1, Follow-up > W1 |
| Sign Language Inst. | 10 | 10 | 68.99 | .000*** | .69 | Pretest-Based: W6 > Pre-test, W7 > Pre-test, Follow-up > Pre-test, W8 > Pre-test, Post-test > Pre-test Development-Based: W6 > W1, W7 > W1, W8 > W1, Post-test > W1, Follow-up > W1 |

## Supplementary Table 20: *Main Idea Component Time-Dependent Progress Analysis (Friedman Test)*

| Instruction Type | n | df | χ² | p | ε² | Significant Differences (Bonferroni Corrected p < .05) |
| --- | --- | --- | --- | --- | --- | --- |
| Guided Reading | 10 | 10 | 82.95 | .000*** | .83 | Development/Posttest-Based: Post-test > W1, Follow-up > W1, W8 > W1 Post-test > W2, Follow-up > W2, W8 > W2 Post-test > W3, Follow-up > W3, W8 > W3 Post-test > W4, Follow-up > W4, W8 > W4 |
| Dialogic Reading | 10 | 10 | 81.37 | .000*** | .81 | Pretest-Based: W5 > Pre-test, W6 > Pre-test, W7 > Pre-test, W8 > Pre-test, Post-test > Pre-test, Follow-up > Pre-test Development-Based: W5 > W1, W6 > W1, W7 > W1, W8 > W1, Post-test > W1, Follow-up > W1 |
| Sign Language Inst. | 9 | 10 | 60.83 | .000*** | .68 | Pretest/Development-Based: Follow-up > Pre-test, Post-test > Pre-test W8 > W1, Follow-up > W1, Post-test > W1 |

## Supplementary Table 21: *Total score Time-Dependent Progress Analysis Pairwise results*

| Comparison | Test Statistic | Std. Error | Std. Test Statistic (z) | padj (Bonferroni) |
| --- | --- | --- | --- | --- |
| PRETOP – TOP6 | 5.45 | 1.483 | 3.674 | .013* |
| PRETOP – TOP7 | 6.8 | 1.483 | 4.585 | < .001*** |
| PRETOP – FTOP | -7.95 | 1.483 | -5.36 | < .001*** |
| PRETOP – TOP8 | 8.75 | 1.483 | 5.899 | < .001*** |
| PRETOP – POTOP | -9.25 | 1.483 | -6.236 | < .001*** |
| TOP1 – TOP7 | -6.05 | 1.483 | -4.079 | .002** |
| TOP1 – FTOP | -7.2 | 1.483 | -4.854 | < .001*** |
| TOP1 – TOP8 | -8 | 1.483 | -5.394 | < .001*** |
| TOP1 – POTOP | -8.5 | 1.483 | -5.731 | < .001*** |
| TOP2 – FTOP | -6 | 1.483 | -4.045 | .003** |
| TOP2 – TOP8 | -6.8 | 1.483 | -4.585 | < .001*** |
| TOP2 – POTOP | -7.3 | 1.483 | -4.922 | < .001*** |
| TOP3 – TOP8 | -5.4 | 1.483 | -3.641 | .015* |
| TOP3 – POTOP | -5.9 | 1.483 | -3.978 | .004** |
| TOP4 – POTOP | -4.95 | 1.483 | -3.337 | .047* |
